# Collateral Development in Patients Undergoing Coronary Angiography in a Tertiary Care Centre: A Descriptive Cross-sectional Study

**DOI:** 10.31729/jnma.7542

**Published:** 2022-08-31

**Authors:** Ajeevan Gautam, Bishnu Mani Dhital, Manas Pokhrel, Amrit Kafle

**Affiliations:** 1 Department of Anatomy, Chitwan Medical College, Bharatpur, Chitwan, Nepal; 2 Department of Cardiology, Chitwan Medical College, Bharatpur, Chitwan, Nepal; 3 Department of Emergency Medicine, Chitwan Medical College, Bharatpur, Chitwan, Nepal; 4 Department of Internal Medicine, Chitwan Medical College, Bharatpur, Chitwan, Nepal

**Keywords:** *angiography*, *artery*, *blood circulation*, *prevalence*

## Abstract

**Introduction:**

Coronary artery disease is one of the major cardiovascular diseases affecting the global human population. When the primary stenotic or blocked channel fails to deliver enough blood to the myocardium, the coronary collateral circulation serves as a backup source of blood supply. The aim of this study was to find out the prevalence of collateral development in patients undergoing coronary angiography in a tertiary care centre.

**Methods:**

This descriptive cross-sectional study was conducted among patients with peripheral vascular injuries that underwent operative management in a tertiary care centre from 1 November 2021 to 15 April 2022. Ethical approval was taken from the Institutional Review Committee (Registration number: CMC-IRC/078/079-027). Convenience sampling technique was used. Data for the study was retrieved from operation records of the patients along with their treatment summaries. Point estimate and 95% Confidence Interval were calculated.

**Results:**

Among 170 patients undergoing coronary angiography, 84 (49.41%) (41.6-56.6, 95% Confidence Interval) had developed collateral circulation. The mean age was 62.8±11.7 years.

**Conclusions:**

The prevalence of collateral development in the coronary artery was similar to other published literature.

## INTRODUCTION

One of the most common cardiovascular diseases impacting people worldwide is coronary artery disease (CAD).^1^ The latest World Health Organization (WHO) figures show that 12.26% of all fatalities in Nepal in 2020 were caused by coronary heart disease.^2^ Before there is lasting damage, it is imperative that we revascularize the coronary artery as soon as possible during the "golden time" window.^3^

Collaterals of the coronary artery start developing at birth but the functional capabilities of it vary between the individuals.^4^ Collaterals offer the myocardium threatened by occlusive coronary artery disease a different source of blood flow. In the case of a persistent complete coronary blockage, they assist in maintaining myocardial function.^5^ Different interventions available are percutaneous coronary intervention (PCI) and coronary artery bypass grafting (CABG).

The aim of this study was to find out the prevalence of collateral development in patient undergoing coronary angiography at a tertiary care centre.

## METHODS

This descriptive cross-sectional study was conducted among the patients undergoing coronary angiography at Chitwan Medical College from 1 November 2021 to 15 April 2022. Ethical approval was taken from the Institutional Review Committee of Chitwan Medical College (Registration number: CMC-IRC/078/079-027). All patients who had received a diagnosis of coronary artery disease were included. Patients with additional comorbid diseases, patients with a history of ischemic heart disease, and patients who had received a coronary artery bypass graft were excluded from the study. Convenience sampling technique was used. The sample size was calculated using the following formula:


$$\begin{array}{ll} \text{n} & ={\text{Z}}^{2}\times \frac{\text{p}\times \text{q}}{{\text{e}}^{\text{2}}}\\ & =1.96^{2}\times \frac{0.50\times 0.50}{0.08^{2}}\\ & =151 \end{array}$$

Where,

n= minimum required sample size
Z= 1.96 at 95% Confidence Interval (CI)
p= prevalence taken as 50% for maximum sample size calculation
q= 1-p
e= margin of error, 8%

The minimum required sample size was 151 but a sample of 170 was taken. The selection of patients was made via clinical suspicion, examination, electrocardiogram (ECG), and imaging methods. Symptoms of coronary artery disease such as chest pain, pain radiating to the neck or arm that can't be explained by other tests, new or increasing chest pain, and abnormal results on the noninvasive heart stress test.^7^ Coronary collaterals, often known as "natural bypasses," are anastomotic connections between various coronary arteries and within the same coronary artery that do not require a capillary bed in between.^8^ During the procedure the presence of the collaterals was recorded.

Data were entered and analysed in IBM SPSS Statistics 20.0. Point estimate and 95% CI were calculated.

## RESULTS

Among 170 patients, 84 (49.41%) (41.89-56.93, 95% CI) patients had developed collateral circulation during the study period. The study reported left anterior descending (LAD) and right coronary artery (RCA) are the commonest arteries occluded. Complete occlusion of the LAD artery was 15 (17.85%), similarly complete RCA occlusion was seen in 22 (26.19%). In the group of 75-100% stenosis 41 (48.80%) was seen in LAD, similarly 38 (45.23%) in Left circumflex artery (LCX) and 45 (53.57%) in RCA (Table 1).

**Table 1:** Frequency showing the status of coronary artery (n= 84).

Stenosis Category	Coronary artery			
	LAD n (%)	LCX n (%)	LM n (%)	RCA n (%)
100%	15 (17.85)	3 (3.57)	-	22 (26.19)
75-100%	41 (48.80)	38 (45.23)	3 (3.57)	45 (53.57)
50-75%	14 (16.66)	10 (11.90)	1 (1.19)	8 (9.52)
30-50%	14 (16.66)	5 (5.95)	-	2 (2.38)

The collateral development in males was 48 (57.14%) and in females was 36 (42.86%) (Table 2). The mean age of the patients was 62.8±11.7 years.

**Table 2:** Sex distribution study population (n= 84).

Sex	n (%)
Male	48 (57.14)
Female	36 (42.86)

## DISCUSSION

The prevalence of collateral development observed was 49.4% and it has been found in males (54.11%). Normally coronary artery collaterals are seen in 9% of the total population.^6^ In the patient with coronary artery blockage the rate of formation of collaterals increases to 35-95%.^6,9^ Our study shows that the prevalence of collateral formation is 49.40% among the patients undergoing coronary angiography at a tertiary care hospital in Nepal. Ever-increasing coronary artery disease cases are a growing public health concern.^10^ Our study shows male predominance. A similar result has been given by other studies.^11^

A study on predictors of well-developed coronary collateral circulation in a patient with stable angina was conducted in Turkey and reported that collateral development was found in 48.1% of individuals.^1^ Our study also reported that 49.40% of the study population developed collaterals. A study to determine the determinants of collateral development in a patient with acute MI was conducted in Japan and reported 37.1% of the individuals with complete collateral circulation.^13^ Our findings are more (49.4%) than the study done in Japan.

A study in coronary artery disease reported the left anterior descending artery was commonly involved followed by the right coronary artery and left circumflex artery.^14^ The findings of the study are similar to ours. This similarity might be because of a similar pattern, position of the artery, and similar study population. As shown in an investigation done in the United States, collateral circulation was present in 27% of individuals receiving immediate medical attention for acute myocardial infarction.^15^ Our findings are more (49.4%) than the study done in the United States. A study reported collateral circulation was present in 73.07% of the coronary artery.^16^ Our findings are less (49.04%) than the study done in Israel.

This was a single-centred cross-sectional study with limited sample size. Only patients with cardiac problems who were clinically confirmed were taken for the study.

## CONCLUSIONS

The prevalence of collateral development among patients undergoing coronary angiography in our study was similar to other studies done in similar settings. Collaterals can aid to maintain myocardial function in the event of a chronic total coronary blockage by providing an alternative source of blood supply to myocardium that is at risk due to occlusive coronary artery disease.
